# Postoperative complications of pediatric cataract surgery and their comparison between limbal and pars plana approaches: a meta-analysis

**DOI:** 10.5935/0004-2749.2024-0151

**Published:** 2025-02-13

**Authors:** Bing Zhang, Minying Zhu, Yune Zhao

**Affiliations:** 1 Eye Hospital and School of Ophthalmology and Optometry, Wenzhou Medical University, Wenzhou, Zhejiang, China.; 2 National Clinical Research Center for Ocular Diseases, Wenzhou, Zhejiang, China.

**Keywords:** Pediatric cataract surgery, Postoperative complications, Limbal route, Pars plana routes, Meta-analysis

## Abstract

**Purpose:**

To compare the incidence rates of complications following pediatric cataract surgery between the limbal and pars plana approaches.

**Methods:**

PubMed, EMBASE, Web of Science, Scopus, Cochrane Library, and ClinicaITriaIs.gov were systematically searched for studies comparing the two surgical approaches. We pooled the incidence rates of postoperative complications using a random-effects model.

**Results:**

Seven studies comprising 375 eyes from 260 patients were included. No significant differences in complication rates were observed between the limbal and pars plana approaches. The pooled incidence rates (95% confidence Interval) of postoperative visual axis opacity (VAO), VAO treated with laser or surgery, secondary glaucoma, wound leakage, corneal edema, anterior chamber reaction, posterior iris synechiae, capsular phimosis, intraocular lens dislocation, posterior capsular rupture, and intravitreal lens fragmentation were 4.7% (0.8%-10.8%), 3.9% (1.0%-8.1%) , 2.8% (0%-11.4%), 0 (0%-1.3%), 2.9% (0%-11.8%), 5.6% (0.1%-16.5%), 2.4% (0%-8.5%), 3.8% (0.6%-8.9%), 2.2% (0%-6.4%), 9.2% (4.1%-15.8%) and 1.3% (0%-6.3%), respectively. Both surgical approaches demonstrated improved visual acuity postoperatively.

**Conclusions:**

Pediatric cataract surgery, performed via the limbal or pars plana approach, is effective and safe, with a low incidence of complications when conducted by trained surgeons. Neither method demonstrated a significant difference in the visual acuity improvement or complication rates.

## INTRODUCTION

Pediatric cataract, a leading cause of treatable childhood blindness globally^(1)^, affects an estimated 1-6 per 10,000 live births^(1^,^2^,^3)^. Timely intervention of this condition, which often involves cataract removal and intraocular lens (IOL) implantation or other refractive correction measures, is crucial to ensure normal visual development^(4)^. However, postoperative complications, such as visual axis opacity (VAO), can compromise the postoperatively achieved visual function. Posterior capsulectomy and anterior vitrectomy is the current mainstay in the treatment of pediatric cataracts, especially in young children^(5^,^6^,^7)^. Surgical approaches for pediatric cataracts primarily involve either the limbal or the pars plana route. However, studies comparing the two approaches are limited. Both approaches offer distinct advantages, with anterior segment surgeons generally preferring the limbal technique and retinal surgeons often favoring the pars plana route^(8^,^9^,^10)^.The current evidence on postoperative complications in pediatric cataracts is often limited by small sample sizes, particularly when comparing surgical approaches. Thus, we aimed to conduct a meta-analysis to comprehensively assess the postoperative complications associated with pediatric cataract surgery and compare the outcomes of the limbal and pars plana techniques. This comparative analysis addresses a critical knowledge gap that can guide surgical decision-making and inform pediatric surgeon training.

## METHODS

### Search strategy

We conducted a comprehensive search across multiple databases, including PubMed, EMBASE, Web of Science, Scopus, and the Cochrane Library using the following search strategy: (Transcorne* OR Trans-corne* OR Limb* OR Cornea*) AND (“Pars Plana”) AND (Vi-trectom* OR Vitreous) AND (Pediatric OR Congenital OR Infant OR Child* OR Adolescent OR Juvenile* or Minor* OR Inherit*) AND Cataract. Additionally, we searched ClinicalTrials.gov using the following keywords: *Cataract [Condition or disease], pars plana AND (Limbal OR Cornea OR Transcornea) [Other terms].* The search was restricted to English language articles. The search yielded a total of 616 studies from the databases. The references of each study were also searched for additional relevant studies, and three of them were identified. A single study identified on ClinicalTrials.gov was excluded due to its irrelevance to our study.

### Eligibility criteria and study selection

*Inclusion Criteria:* Studies were included if they met the following **PICOS** criteria.

**P**opulation: The study population were patients aged <18 years who were diagnosed with congenital, developmental, infantile, pediatric, juvenile, or inherited cataract.

**I**nterventions and **C**omparisons: The study compared the limbal and pars plana routes for pediatric cataract removal, with or without IOL implantation.

**O**utcomes: The study outcomes were VAO (the primary outcome) and other postoperative complications.

**S**tudy design: The study was a prospective or retrospective cohort study that followed patients’ postoperative outcomes.

*Exclusion criteria:*

Reviews, case reports, and studies that introduced surgical techniques.

*Review process:*

Two reviewers (B.Z. and M.Z.) independently screened the titles and abstracts of the identified studies and subsequently reviewed the full texts of these studies.

Discrepancies were resolved via discussion with a senior reviewer (Y.Z.).

### Data extraction

Two reviewers (B.Z. and M.Z.) extracted the data to minimize errors. Extracted variables included: study characteristics; baseline patient information; follow-up details; surgical procedures for two surgical route groups; intraoperative and postoperative outcomes and complications

### Statistical analysis

All analyses and graph plotting were conducted using STATA (version 17.0, StataCorp LLC, College Station, TX, USA). Meta-analysis of the incidence rates, odds ratios (ORs), and 95% confidence intervals (Cls) were performed when two or more studies reported the same outcome. A random-effects model (DerSimonian-Laird method)^(11)^ was employed due to the assumed heterogeneity between studies. Meta-analyses of the ORs and incidence rates were performed using the *meta* and *metaprop* packages, respectively^(12)^. For reference, the incidence rates with a fixed-effects model were also reported.

## RESULTS

### Literature description

**Figure 1:**
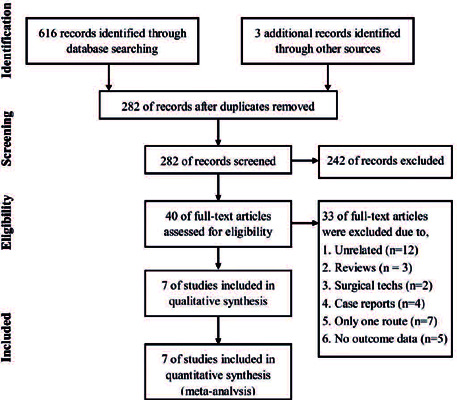
Flow diagram of the study selection process.

Of the 619 identified studies, 337 were duplicates. Of the 282 unique studies, 40 underwent full-text screening. Finally, seven studies were included in the meta-analysis (Figure 1)^(13^,^14^,^15^,^16^,^17^,^18^,^19)^. Table 1 presents the baseline characteristics of the seven studies included in the analysis, reflecting the demographic and clinical variables of the participants at the time of enrollment. A total of 375 eyes of 260 patients (50.5% male) across three randomized controlled trials (RCTs), two non--RCT prospective cohorts, and two retrospective studies were pooled in the analysis. The baseline age of the study participants was comparable between the surgical approaches in most studies, except in one retrospective study^(17)^. The follow-up time was similar between the surgical approaches in most studies, except in the two retrospective studies^(15^, ^17)^.

**Table 1:** Characteristics of the included studies

No.	First author, year	Study design	Location	Patients /eyes	Male, n (%)	Age (months)	Type of cataract	Followup (months)	Limbal Patients/eyes	Pars plana Patients/eyes	p-value for age	p-value followup
1	Ahmadieh H(13), 1999	RCT (double-marked)	Tehran, lran	31/45*	12 (39)	75.6 ± 24	34 development + 4 traumatic	12	19/19	19/19	>0.05	>0.05†
2	Raina UK(14), 2016	RCT (self-control)	New Delhi, India	12/24	7 (58)	7.5 (3-12)	Pediatric	12	12/12	12/12	>0.05	>0.05t
3	Liu X (15), 2016	Retrospective case series	Shanghai, China	56/81	30 (54)	46 (18-72)	Congenital	31 (24-48) vs. 57 (36-77)	26/40	30/41	0.414	<0.001
4	Gawad SA(16), 2019	RCT	Mansoura, Egypt	21/30	12 (57)	12.95 ± 5.65	Congenital	6	15/15	15/15	0.259	>0.05t
5	Koch CR(17), 2019	Retrospective case series	Barcelona, Spain	46/56	NA	34.5 ± 22.66	Pediatric	139.6 ± 36.7 vs. 101.8 ± 28.6	27/27	29/29	<0.001	<0.001
6	Alshamahi E(18), 2021	Prospective (non-RCT)	Sana'a, Yemen	60/71	33 (55)	13.6 ± 13.0	Congenital	46.9 ± 7.4 vs. 42.8 ± 10	22/25	38/46	0.75	0.09
7	Dawoud M(19), 2021	Prospective (non-RCT)	Alexandria/ Menoufia, Egypt	34/68	14 (41)	7.7 ± 4.5	Congenital	6	17/34	17/34	>0.05	>0.05†

* 38 eyes in study 1 were quantitatively analyzed;

† The followup time was the same in the two groups; thus, p > 0.05 was allocated.

### Surgical effects and duration

Two of the included studies assessed the postoperative visual acuity (VA). Liu et al. found no significant difference in the best-corrected VA (BCVA) at baseline and the last follow-up between the two surgical approaches^(15)^. Specifically, the mean postoperative logMAR BCVA was 0.32 (range, 0-1.3) in the limbal group and 0.35 (range, 0-1.3) in the pars plana group (p = 0.642)^(15)^. Ahmadieh et al. categorized the postoperative VA improvement as a 4-level hierarchical variable, and there was no significant difference in VA improvement between the two surgical routes^(13)^.

Two studies compared the operative time of the two surgical approaches. Both studies demonstrated significantly shorter operative times in the limbal group than in the pars plana group. The operative times were 39.93 ±.04 and 59.27 ± 12.69 mins in the limbal and pars plana groups, respectively, in one study in which cataract removal and lOL implantation were performed together (p<0.001)^(16)^. ln the other study, the operatives times for cataract removal alone were 9.8 ± 0.9 and 16.5 ± 3.3 mins (p<0.001) in the limbal and pars plana groups, respectively^(19)^.

### Postoperative complications

**Figure 2:**
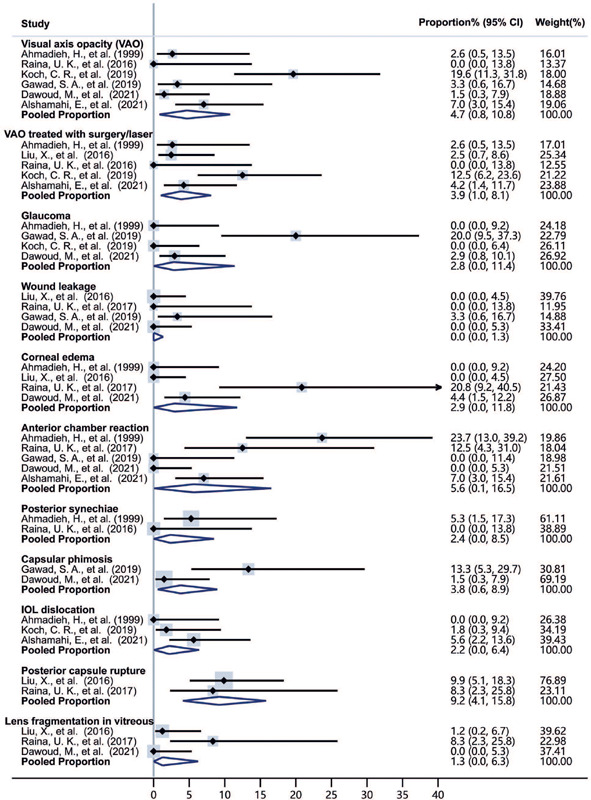
Forest plots of the pooled rates of the surgical complications.

Figure 2 shows the pooled incidence rates of different complications using the random-effects model. The pooled rate of VAO was 4.7% (95% Cl, 0.8%-10.8%), and the pooled rate of VAO with secondary surgery or laser was 3.9% (95% Cl, 1.0%-8.1%). The pooled incidence rates of only anterior chamber reaction (5.6%, 95% Cl 0.1%-16.5%) and posterior capsule rupture (9.2%, 95% Cl 4.1%-15.8%) exceeded 5%, which is considered the significant cutoff point for small probability events.

**Figure 3:**
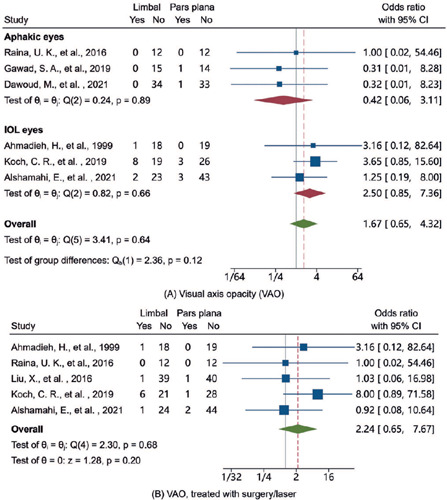
Forest plots comparing the VAO rates between the limbal and pars plana approaches. (A) VAO rates stratified by the state of IOL implantation. (B) Rates of VAO treated with secondary surgery or laser.

**Figure 4:**
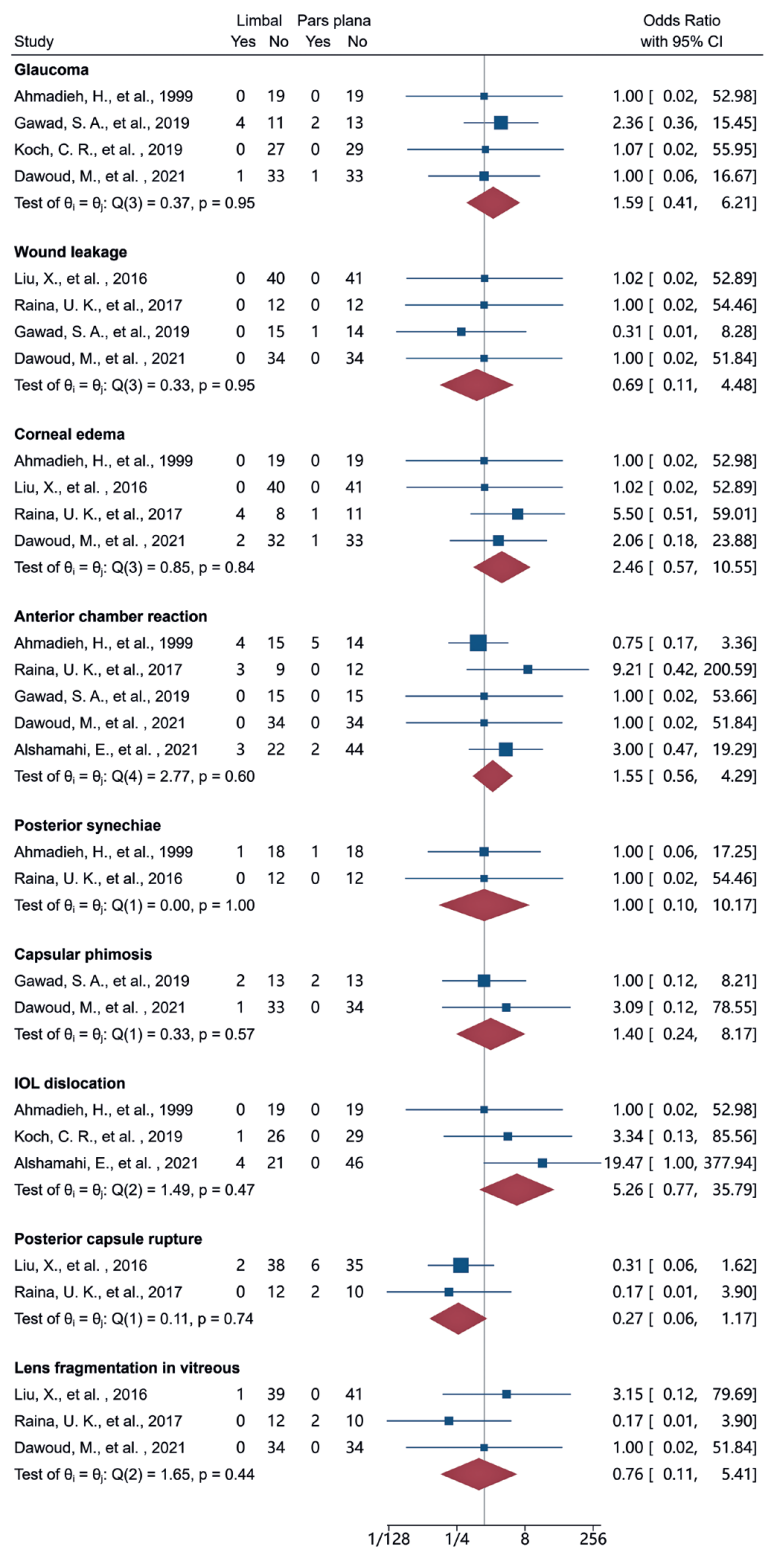
Forest plots comparing the surgical complication rates between the limbal and pars plana approaches.

Table 2 shows the incidence rates of the complications stratified by the two surgical approaches. The incidence rates based on the random-effects model are presented and the pooled incidence rates of VAO, treated VAO, secondary glaucoma, corneal edema, anterior chamber reaction, capsular phimosis, and lOL dislocation were higher in the limbal group than in the pars plana group. Conversely, the pooled incidence rates of wound leakage, posterior capsule rupture, and intravitreal lens fragmentation were higher in the pars plana group than in the limbal group. Figures 3 and 4 depict the pooled ORs of the complications between the two surgical approaches, which were calculated using the DerSimonian-Laird method. There were no significant differences in any postoperative complication between the two surgical approached. The ORs for VAO were not significantly different between the limbal and pars plana groups in aphakic eyes (OR, 0.42; 95% Cl, 0.06-3.11), IOL eyes (OR, 2.5; 95% Cl, 0.85-7.36) or all pooled eyes (OR, 1.67; 95% Cl, 0.65-4.32). Similarly, there was no significant difference in the OR for VAO treated with laser or secondary surgery between the two surgical approaches (OR, 2.24; 95% Cl, 0.65-7.67). The ORs for the other complications are presented in figure 4. There were no significant differences in any complications between the surgical routes. The pooled ORs (limbal versus pars plana route, 95% Cl) for secondary glaucoma, wound leak, corneal edema, anterior chamber reaction, posterior synechiae, capsular phimosis, IOL dislocation, posterior capsule rupture, and intravitreal lens fragmentation were 1.59 (0.41-6.21), 0.69 (0.11-4.48), 2.46 (0.57-10.55), 1.55 (0.56-4.29), 1.00 (0.10-10.17), 1.40 (0.24-8.17), 5.26 (0.77-35.79), 0.27 (0.06-1.17), and 0.76 (0.11-5.41), respectively.

**Table 2:** Pooled rates of different complications stratified by surgical approaches.

Complication	Enrolled studies	Limbal group				Pars plana group				All			
		Pooled events/ eyes	l^2^%	Pooled rate, % (95% CI)		Pooled events/ eyes	l^2^%	Pooled rate, % (95% CI)		Pooled events/ eyes	l^2^%	Pooled rate, % (95% CI)	
				Fixed	Random			Fixed	Random			Fixed	Random
VAO	6	11/132	72.0	4.9 (1.4, 10)	4.6 (0, 15.2)	8/155	0	4.0 (1.0, 8.3)	4 (1, 8.3)	19/287	69.7	5.2 (2.7, 8.3)	4.7 (0.8, 10.8)
VAO treated with surgery/ laser	5	9/123	49.4	5.6 (1.7, 11)	5.6 (0.6, 13.8)	4/147	0	2.0 (0.1, 5.6)	2 (0.1, 5.6)	13/270	45.2	4.0 (1.7, 6.9)	3.9 (1, 8.1)
Glaucoma	4	5/95	69.1	2.6 (0, 7.6)	3.4 (0, 14.7)	3/97	33.0	1.6 (0, 6)	1.8 (0, 7.7)	8/192	80.2	2.1 (0.3, 5)	2.8 (0, 11.4)
Wound leakage	4	0/101	0	0 (0, 1.7)	0 (0, 1.7)	1/102	0	0 (0, 2.7)	0 (0, 2.7)	1/203	0	0 (0, 1.3)	0 (0, 1.3)
Corneal edema	4	6/105	76.5	2.5 (0, 7.2)	4.4 (0, 18)	2/106	7.4	0.7 (0, 4.2)	0.7 (0, 4.5)	8/211	81.6	1.7 (0.1, 4.3)	2.9 (0, 11.8)
Anterior chamber reaction	5	10/105	72.7	5.9 (1.6, 12)	7.6 (0.1, 21.7)	7/126	66.6	2.7 (0.2, 7.1)	3 (0, 12.3)	17/231	84.5	4.6 (2, 8)	5.6 (0.1, 16.5)
Posterior synechiae	2	1/31	0	2.3 (0, 12.6)	2.3 (0, 12.6)	1/31	0	2.3 (0, 12.6)	2.3 (0, 12.6)	2/62	0	2.4 (0, 8.5)	2.4 (0, 8.5)
Capsular phimosis	2	3/49	0	5.1 (0.2, 14)	5.1 (0.2, 14)	2/49	0	1.5 (0, 8.3)	1.5 (0, 8.3)	5/98	0	3.8 (0.6, 8.9)	3.8 (0.6, 8.9)
IOL dislocation	3	5/71	57.1	5.3 (0.7, 12.5)	5.0 (0, 16.7)	0/94	0	0 (0, 2)	0 (0, 2)	5/165	35.6	2.4 (0.4, 5.7)	2.2 (0, 6.4)
Posterior capsule rupture	2	2/52	0	2.8 (0, 10.3)	2.8 (0, 10.3)	8/53	0	14.5 (5.7, 26)	14.5 (5.7, 26)	10/105	0	9.2 (4.1, 15.8)	9.2 (4.1, 15.8)
Lens fragmentation in the vitreous	3	1/86	0	0.4 (0, 4.1)	0.4 (0, 4.1)	2/87	66.2	0.3 (0, 3.7)	1.2 (0, 10.9)	3/173	59.0	0.8 (0, 3.2)	1.3 (0, 6.3)

## DISCUSSION

Of the 282 identified unique records, seven studies encompassing 375 eyes of 260 pediatric patients with cataract were analyzed in this systematic review. The primary objective of the study was to compare the incidence rates of complications following pediatric cataract surgery between the limbal and pars plana approaches.

### Overall safety and efficacy

The pooled analysis revealed favorable outcomes following both surgical approaches, with low incidence rates of various complications following cataract removal with anterior vitrectomy. These results support the safety of this strategy for managing pediatric cataracts. Almost all the pooled incidence rates were near or below 5%, a commonly used threshold for small probability events. Only two of the seven included studies reported outcomes on the postoperative VA^(13^,^15)^, which may reflect the challenge in assessing VA in young children. Although it was impossible to pool the VA data due to differences in measurement methods, both studies demonstrated an improvement in postoperative VA and similar surgical effects between the two approaches^(13^, ^15)^. Evidence suggests that both approaches can improve VA. Furthermore, the similar VA outcomes may be attributed to the achievement of a clear visual axis, which is a critical factor in restoring visual function in pediatric patients with cataract^(20)^.

Two studies compared the operative times of the pars plana and limbal approaches. The operative time via the pars plana approach was significantly longer than that via the limbal approach for cataract surgery with^(16)^ or without^(19)^ lOL insertion.

### Complications

The most prevalent complication following pediatric cataract surgery appeared to be posterior capsule rupture, with a pooling rate of 9.2% (95% Cl, 4.1%-15.8%). However, this incidence is based on the data from only two studies that evaluated 105 eyes. The posterior capsule’s thinner structure and potential defects can make pediatric cataract surgery more challenging^(21)^. Consequently, posterior capsulectomy is often a demanding task for surgeons. Although not significantly significant, posterior capsule tears were more frequent with the pars plana approach than with the limbal approach, necessitating increased surgeon vigilance. The second most prevalent complication is anterior chamber reaction, with a pooled rate of 5.6% (95% Cl, 0.1%-16.5%). This complication is more common with the limbal approach than with pars plana approach due to more anterior chamber operations. Based on our experience, anterior chamber reactions can be managed in most cases with appropriate topical anti-inflammatory agents. VAO is a significant postoperative complication of pediatric cataract surgery that can obstruct the visual axis and potentially lead to visual impairment. The pooled incidence of VAO in the study was 4.7% (95% Cl, 0.8%-10.8%). The pars plana technique is generally considered more effective in achieving complete capsulectomy, lens removal, and anterior vitrectomy^(9^,^13^,^15)^. Thus, some authors believe that VAO may be more common with the limbal approach than with the pars plana approach. Although the difference was statistically insignificant, in the lOL eyes, the pooled incidence of VAO was higher in the limbal group than in the pars plana group. However, in the aphakic eyes, the incidence of VAO was lower in the limbal group than in the pars plana group. This might be attributed to the better visualization of intraocular structures with the limbal approach, facilitating complete lens removal. Conversely, for lOL implantation, the pars plana technique may offer advantages over the limbal approach for achieving adequate capsulectomy and vitrectomy^(9^,^13)^. Secondary glaucoma is another important complication of pediatric cataract surgery, with a pooled rate of 2.8% (95% Cl, 0%-11.4%). The definition of glaucoma varied across the included studies in our review. Among the four studies reporting secondary glaucoma^(13^,^16^,^17^,^19)^, only one defined it as an intraocular pressure >21 mmHg with changes in the cup-to-disc ratio^(17)^. Other complications are rare. Wound leakage developed in only one eye in the pars plana group^(16)^. The incidence rates of corneal edema, posterior iris synechia, lOL dislocation, and intravitreal lens fragmentation were <3%, indicating a low occurrence of these events.

### Limitations

This review has several limitations. First, only three of the seven studies were RCTs, and the outcomes in the non-RCTs may have been influenced by differences in the baseline characteristics between the two surgical groups. Second, some complications, such as glaucoma, were not clearly defined in most studies. Third, the patient age and followup duration varied among the included studies. However, within each individual study, the patient age and followup duration were generally comparable between the surgical approach groups.

In conclusion, our review and meta-analyses suggest that cataract surgery is an effective and safe treatment for pediatric patients with cataracts. Furthermore, cataract surgery is associated with improved postoperative VA and low complication rates. However, neither surgical method demonstrated a significant difference in VA improvement or complication rates.
